# High-Throughput Sequencing Application in the Diagnosis and Discovery of Plant-Infecting Viruses in Africa, A Decade Later

**DOI:** 10.3390/plants9101376

**Published:** 2020-10-16

**Authors:** Jacques Davy Ibaba, Augustine Gubba

**Affiliations:** Discipline of Plant Pathology, School of Agricultural, Earth and Environmental Sciences, Agriculture Campus, University of KwaZulu-Natal, Scottsville, Pietermaritzburg 3209, South Africa; GubbaA@ukzn.ac.za

**Keywords:** plant virology, food security, Illumina HiSeq, Illumina MiSeq, 454 pyrosequencing, Maize, common bean, cowpea, sweet potato, cassava

## Abstract

High-throughput sequencing (HTS) application in the field of plant virology started in 2009 and has proven very successful for virus discovery and detection of viruses already known. Plant virology is still a developing science in most of Africa; the number of HTS-related studies published in the scientific literature has been increasing over the years as a result of successful collaborations. Studies using HTS to identify plant-infecting viruses have been conducted in 20 African countries, of which Kenya, South Africa and Tanzania share the most published papers. At least 29 host plants, including various agricultural economically important crops, ornamentals and medicinal plants, have been used in viromics analyses and have resulted in the detection of previously known viruses and novel ones from almost any host. Knowing that the effectiveness of any management program requires knowledge on the types, distribution, incidence, and genetic of the virus-causing disease, integrating HTS and efficient bioinformatics tools in plant virology research projects conducted in Africa is a matter of the utmost importance towards achieving and maintaining sustainable food security.

## 1. Introduction

Viruses were found to cause diseases in plants in the last quarter of the 19^th^ century. This discovery, which also marked the start of the discipline of plant virology, was made from various studies that pinpointed tobacco mosaic virus as the causal agent of a mosaic disease on tobacco plants [1]. Thenceforth, viruses have been identified in most plants including vegetables [2,3], legumes [4,5], cereals [6,7], fruit crops [8], ornamentals [9] and wild species [10] and constitute approximately one-third of plant disease-causing agents. Plant virus particles, frequently referred to as virions, vary in shapes and sizes. They are obligatory intracellular parasites made of a single or multiple DNA or RNA genomic segments enclosed within a protein shell called the capsid. Most plant-infecting viruses have non-enveloped virions except the members of families *Firmoviridae* [11], *Rabdoviridae* [12] and *Tospoviridae* [13]. The host plant cuticle and the cell wall provide a solid natural physical protection that has to be broken to create virus entry point to the plant cell of a susceptible host and cause disease. This is generally achieved through mechanical wounds or the action of vectors such as insects, nematodes, fungi while feeding on the plants. Following the entry into a host cell and genome decapsidation, the infectious cycle includes translation and replication of the viral genome, assembly of progeny virus particles, generalized invasion of the host through cell-to-cell and long-distance movements of viral particles or ribonucleoprotein complexes and finally, transmission to new hosts by vectors [14]. Virus transmission also occurs through the use of infected plant propagation materials, grafting and contaminated tools. 

Plant-infecting viruses are of serious concern in agriculture and represent a significant threat to food security worldwide. Virus infection in susceptible host plants results in a series of physiological disorders that have adverse effects on the overall plant heath [14]. The symptoms typical of virus infections are quite diverse and may appear on any part of the infected plants. The severity of these symptoms results from interactions between the virus, the host plant, the virus vector and the environment [15,16]. The family *Potyviridae*, the largest family of RNA plant-infecting viruses [17] and the second-largest plant virus family after *Geminiviridae* [18], comprise some of the most damaging and widespread viruses of agronomic crops worldwide. In some instances, over 90% crop yield reduction has been recorded [14]. Such losses have had devastating socio-economic consequences for farmers, producers, distributors and consumers.

Accurate diagnosis of virus diseases is crucial for developing effective and sustainable crop management systems [19], especially considering that several biotic and abiotic factors also produce virus-like symptoms in plants [20,21]. The use of appropriate integrated control strategies upon virus identification will prove effective in mitigating the spread of the virus, thereby reducing further crop damage and yield loss. The other aspect of integrated management approaches is to prevent the dissemination of virus causing diseases through their different modes [22]. One of the ways to achieve this goal has been using certified high-quality virus-tested planting materials. However, the large number and high genetic variability of plant viruses can make their detection cumbersome [23]. 

The methods of detection and identification of viruses developed heretofore fall into serological techniques, molecular methods, microscopical and physical observations [19,20]. Serological assays such as the enzyme-linked immunosorbent assay and the tissue blot immunoassay, and the polymerase chain reaction and its derivatives from the molecular side, have been commonly used for routine testing [20,21,24]. High sensitivity, specificity, reliability, speed, and cost effectiveness are some of the determinant factors of the success of any method of detection [22,24]. High-throughput sequencing (HTS) has been gaining popularity as a powerful alternative for diagnosis and detection of plant viruses worldwide. This review focuses on the contribution of HTS in Africa in relation to the detection and discovery of plant-infecting viruses based on the peer-reviewed articles published since the beginning of HTS applications in plant virology. 

## 2. Integration of HTS in Plant Virology in Africa

HTS was initially introduced as next generation sequencing to mark a distinct demarcation from the former sequencing technology commonly known as Sanger sequencing that was introduced in 1977. HTS technologies employ unprecedented parallelization of a high number of sequencing reactions, generating billions of reads that amount to gigabytes of data, in a shorter time and at a much-reduced cost [25]. Either digital single or pair-end reads can be obtained depending on the technology selected. Since their introduction in 2005, many HTS sequencing platforms have been developed including Pyrosequencing, Ion Torrent technology, Illumina/Solexa, and Sequencing by Oligonucleotide Ligation and Detection. Further optimization has led to innovative third- and fourth-generation platforms such as single-molecule real-time sequencing by PacBio and Nanopore sequencing [26]. These platforms differ substantially in terms of their preparatory procedures, sequencing chemistry or technology, nucleotide incorporation technology, read type, length, accuracy, the size of generated data and price. More elaborate information on HTS can be found from previous reviews [25,26,27,28,29,30,31]. 

The application of HTS combined with the rapid progress in developing bioinformatics tools to simplify data analysis has revolutionized plant virology worldwide. The field of plant virus discovery and diagnosis especially have benefited the most from these technologies with the earliest report dated in 2009 [32,33]. HTS, unlike conventional serological and molecular methods, does not require any knowledge of the suspected causing disease pathogen before being performed. This unique property of unbiased and hypothesis-free testing of plant sample has been the principal reason behind integrating HTS as a method for simultaneous detection of multiple viruses regardless of their genome nature or structure [34]. Consequently, a number of HTS protocols targeting nucleic acid pools such as virion-associated nucleic acids, double-stranded RNAs, total RNAs, ribosomal-RNA-depleted RNAs, messenger RNAs, or small interfering RNAs have been developed concurrently with specific bioinformatics workflows for this purpose [35,36,37,38,39].

The science of plant virology is still in its infancy in several countries across Africa. Only a few countries are reputed to have teaching institutions and modern research facilities. Moreover, Africa as a whole still lags behind developed countries in adopting HTS technologies and has to overcome the shortage of human resources, the limited expenditure in research and equipment, slow internet connectivity, and infrastructural challenges in order to be “omics” ready [40,41,42]. Nonetheless, countries such as South Africa, Kenya, Senegal, Ghana and Nigeria have gained worldwide recognition for establishing HTS platforms and bioinformatics capacities [43,44]. 

**Figure 1:**
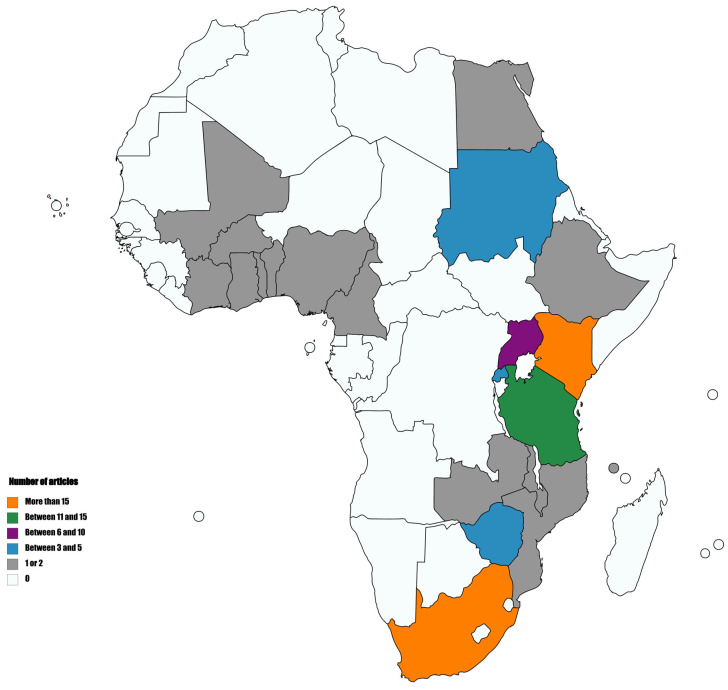
African countries that have been mentioned in high-throughput sequencing (HTS) studies related to the detection and diagnosis of plant-infecting viruses.

On the matter of the application of HTS to the detection and diagnosis of plant-infecting viruses in Africa, a considerable number of studies, made possible mostly through various collaborations with institutions abroad, have been documented. For clarity, the taxonomic classification and abbreviations of viruses detected using HTS in Africa are indicated in Table 1, and a comprehensive summary of these studies is provided in Table 2. The number of peer-reviewed publications around this theme to date stands just above 60 in total. It is important to note that this number has been increasing over the years with a minimum of 10 articles being published starting in 2018. As a point of precision, the African countries as per the African Union list and classification (https://au.int/en/member_states/countryprofiles2) were considered for this review. Twenty countries, out of the 55 that constitute the African continent, have been mentioned in literature on the subject with studies conducted in South Africa, Tanzania and Kenya accounting for more than half of the published articles (Figure 1). South Africa has been the first African country to have locally performed HTS and data analysis related to plant-infecting viruses. Kenya followed some years later. In terms of sequencing platforms, Illumina has been the preferred one for the vast majority of projects. Pyrosequencing platforms have also been used occasionally (Table 2). Satisfactory results were obtained for each study conducted irrespective of the host plant used. Moreover, the choice of the host plants in the HTS related studies in Africa may be closely related to its economic value.

## 3. Host Plants Subjected to HTS

**Figure 2:**
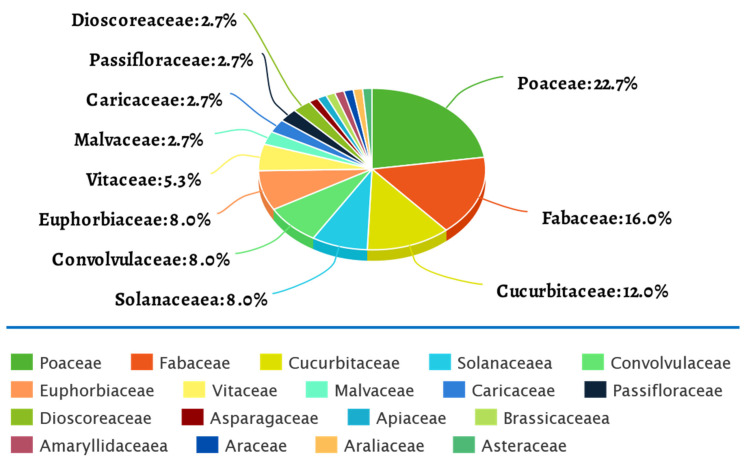
Host plants families that have been subjected to HTS for detection or diagnosis of plant-infecting virus.

Twenty-nine host plants, including crops, medicinal and ornamental plants (Table 2) have been used in HTS for virus detection or diagnostic in Africa since 2010. These host plants belong to 18 different families of which five—*Poaceae*, *Fabaceae*, *Cucurbitaceae*, *Solanaceae, Convolvulaceae* and *Euphorbiaceae*—took the biggest share (Figure 2), mainly because they contain many economically important crops. While some studies focused on a host plant in a specific country or environment as in the case of papaya [52,53], groundnut [69,83], tannia [74], cabbage [96], cotton [103], grapevine [105,106,113,115] to mention a few; in other studies, the viromics of a host plant was analyzed across different countries in a same survey. The information generated in such analyses has provided a better understanding of the status and distribution of the viruses identified for a specific host plant. Selected case studies will be the focus of the next paragraphs.

### 3.1. Maize

Maize, Zea mays L., which originated in the Mexican Highlands, has become one of the major staple food crops in most sub-Saharan African countries, being cultivated in both commercial and small-scale farms. It is also important as feed for poultry and other livestock industries [116]. However, its production over the years has remained lower when compared to the average yield worldwide [117]. 

The first published studies of maize being subjected to HTS dates back to 2013 [112]. HTS was used to elucidate the pathogens causing a new disease of maize, which was first identified in Kenya in 2011. Previous studies of the etiology of this particular disease using ELISA had been unsuccessful. The analysis of the data generated from the sequencing of total RNA on a pyrosequencing platform led to the identification of two viruses, MCMV and SCMV, a combination previously reported to cause maize lethal necrosis disease (MLND). Moreover, the recovery of near-complete genome sequences of the identified virus isolates allowed for their molecular characterization. The MCMV isolate detected, although similar to other previously sequenced strains, was found to be most similar to a Chinese isolate rather than the more widespread American strains based on the complete genome and the translated protein sequences [112]. The SCMV isolate was found to be closely related to a distinct and highly virulent East Asian strain previously found in China, Vietnam and Thailand [112]. MLND is devastating to maize production with losses in East Africa estimated between 25% and 100%. 

MLND symptoms were first observed on maize cultivated in Rwanda in 2013. Four maize samples were subjected to HTS to confirm the presence of the viruses associated with the disease, especially after real-time PCR tested negative for the detection of SCMV. The two viruses responsible for MLND previously identified in Kenya were recovered from the analysis of the HTS data. MCMV was closely related to the MCMV from Kenya and China (99% homology) when compared with MCMV from the United States (96–97% homology). Complete SCMV genomes were found in three of the four samples. The SCMV isolated from the Rwandan samples were distinct from that isolated in Kenya (87% identity). Closer examination of the Rwandan SCMV genomes revealed a high degree of divergence as the cause of the negative real-time PCR results [109]. MCMV and SCMV were also the two viruses detected in a similar study that was conducted in Ethiopia on six maize samples. Phylogenetic trees were constructed based on the complete genomes of MCMV and the coat proteins of the SCMV isolates. MCMV isolates in Ethiopia were found to be highly similar to those found previously in East Africa. SCMV isolates from Ethiopia, in contrast, were found to be similar to each other and those found in Rwanda, but relatively distant from those originally found in Kenya [108]. 

Wamaitha et al. [88] followed a metagenomics analysis based on RNA sequencing, *de novo* assembly and virus identification to gain insight into viruses associated with MLND in Kenya. The virus survey was extended to sorghum and Napier grass. This time, ScaMV, Hubei Poty-like virus 1, JGMV, MYDV-RMV, Barley virus G, MSV were identified in addition to MCMV and SCMV. Based on the virus prevalence and geographic distribution, four viruses—MCMV, SCMV, MSV and MYDV-RMV—were widespread with MYDV-RMV, always found as part of a complex that included MCMV and SCMV, or MCMV, SCMV and MSV. Stewart et al. [118] also confirmed the involvement of JGMV in MLND in Kenya and Uganda.

The prevalence of poleroviruses infecting maize in East Africa was demonstrated by Massawe et al. [87] from the studies conducted on maize growing areas in Kenya, Uganda, Rwanda, and Tanzania. RNA sequencing and *de novo* assembly of non-maize sequences yielded contigs that were aligned to plant-infecting viral sequences in the National Center for Biotechnology Information database. Polerovirus matching contigs were subsequently assembled to generate a supercontig consensus sequence that was further ascertained using primer walking followed by rapid amplification of cDNA ends. The complete genome sequence of the MYDV-like polerovirus isolated from East African countries was 5641 nucleotides and similar to other maize, sugarcane, itch grass-, and barley-associated polerovirus. The prevalence of this virus was estimated between 40.3 and 90.0% [87]. Five virus species not previously reported in Tanzania were reported in 2019 from HTS performed in one of the South African facilities using ribo-depleted RNA [56,57,58,59,60]. One of them, MYMV had a broad geographical distribution and was thought to be another MLND-associated virus. MDMV, SrMV, MMaV, and MATV, although found restricted to specific areas in Tanzania, have already spread throughout East Africa as per the results obtained from HTS recently performed on MLND maize samples collected in Rwanda [55].

### 3.2. Common Bean and Cowpea

Common bean (*Phaseolus vulgaris* L.) and cowpea (*Vigna unguiculata* Walp.) are some African indigenous vegetables that belong to the Family *Fabaceae*. These two crops play an important role in both human nutrition, food security and income generation for farmers and food vendors. The 2016 global estimates showed that 12.3 million hectares of land are utilized in the production of cowpea with Western and Eastern Africa [119]. Three East African countries, Kenya, Tanzania and Uganda, are among the global leaders of common bean production [120].

Seed-borne pathogens, including certain viruses, have great potential to reduce growth and yield of common bean because they interfere with plant growth from the beginning [92]. Against that background, seed-borne viruses were surveyed in common bean varieties, landraces and improved common bean varieties that are grown in Tanzania. The testing was obtained from the seeds of symptomatic plants that had been germinated in insect-proof controlled environments. HTS of the small RNAs extracted from the sampled plant materials were performed. VirusDetect [121], an automated bioinformatics pipeline, was used to detect contigs of viral origins. CPMMV was the only pathogenic virus identified among two other non-pathogenic viruses namely PvEV-1 and PvEV-2. These endornaviruses had not been previously detected on the African continent [92].

Another viromics study of common bean published by Mwaipopo et al. [86] provided a different picture. HTS of small RNAs from leaves sampled, this time, from fields across Tanzania followed by data analysis also performed using VirusDetect led to the detection of several viruses belonging to eleven genera. Apart from the cryptic viruses and CPMMV detected by Nordenstedt et al. [92], BCMNV, BCMV, CABMV, SBMV, CMV, ToLCU-related begomovirus and related umbraviruses were detected.

Mulenga et al. [45] published the results of a comprehensive study of virus-infecting common bean conducted in the Eastern Province of Zambia. HTS was done in South Africa using total RNA. Blastn was subsequently used to query GenBank with the *de novo*-assembled contigs. The detected viruses included SBMV, BCMV, BCMNV, CABMV, and CMV. Out of these viruses, SBMV had never been reported to infect common bean in Zambia. First-generation sequencing of RT-PCR products generated using SBMV specific primers were subsequently conducted to validate the HTS results, thus confirming the incidence of this virus in Zambia.

Kenya is another East African country where HTS has been used for virus identification purposes on common bean. Four studies have been published between 2018 and 2019. Two of these, by Wainaina et al. [71] and Mutuku et al. [84] yielded several viruses while the other two focused on BCMNV [70,72] and CABMV [72]. Wainaina et al. [71] carried out a viral metagenomic analysis using rolling circle amplification to detect possible DNA infecting viruses on symptomatic bean plants. Two approaches were used to identify potential viral sequences within the bean DNA-Seq reads following the quality control check of the reads. Kaiju [122], a program for sensitive taxonomic classification of HTS reads was the method used to determine the taxonomic profile which was visualized in Krona [123]. The other approach consisted in subjecting assembled contigs to blastx searches using a preassembled viral NCBI RefSeq database. PVBV was the only plant-infecting DNA virus detected. Surprisingly some contigs matched BCMNV, a positive single-strand RNA virus [71]. Mutuku et al, [84], on the other hand, utilized shotgun RNA sequencing for metagenomic examination of viruses present in bean plants growing at two locations in Kenya. *De novo* assembly and blast (https://blast.ncbi.nlm.nih.gov/Blast.cgi) were performed and led to the detection of BCMNV, PvEV1, PvEV2, and CMV. RT-PCR was subsequently done to authenticate the presence of these viruses in the respective samples. Molecular analysis of the CMV strain detected was found most closely related to Asian strains that might have been recently introduced to the region. The detection of PvEV1 and PvEV2 suggested that these seed transmitted viruses may be more prevalent in Eastern African bean germplasm than previously thought [84].

A virion-associated nucleic acid (VANA)-based metagenomics method was used to screen for the presence of cowpea infecting viruses in Burkina Faso, a West African country [104]. The VANA were extracted from plants displaying virus-like symptoms collected from the three agro-climatic zones of the country. The sequencing was carried on a pyrosequencing platform. *De novo* assembly of cleaned reads was the method opted and the generated contigs and non-assembled reads with a minimum length of 45 bp were compared to sequences in the GenBank database using blastn and blastx. The presence of viruses identified during the metagenomic screen was verified using RT-PCR. Only RNA viruses were identified which included previously reported viruses such as CABMV, BCMV-BlCM, CPMoV and novel virus species provisionally named CPPV-1, CPPV-2, CPTV-1, CPTV-2 and CPaMV-1 [104].

### 3.3. Sweet Potato

Sweet potato (*Ipomoea batatas* [L.] Lam) is a dicotyledonous plant that belongs to the family *Convolvulaceae*. It has become an important staple and co-staple food crop in Africa. Orange-fleshed sweet potato varieties, a rich source of vitamin A especially, are important for infants and young children. Africa was the second-largest producer, producing 21 million tons in 2016, behind Asia’s production that was estimated at more than 78 million in the same year [124].

Virus diseases have been identified as the second-most important biotic constraint to sweet potato production. A viral metagenomic approach was adopted in South Africa to understand the progressive deterioration of the yield and quality of sweet potato crops, usually referred to as “cultivar decline” experienced by farmers across the country. Leaf samples collected from different surveys in the major growing regions were subjected to DNA [62,79,80], total RNA [80] and small RNAs extraction [81]. Before HTS, the extracted RNA was depleted of ribosomal RNA while rolling circle amplification was performed on the DNA. Good quality reads were used in both *de novo* and reference-guided assemblies. The results indicated the presence of two badnaviruses, sweet potato badnavirus A (SPBVA) and sweet potato badnavirus B (SPBVB), which had never been reported [81] along with commonly occurring viruses, i.e., SPFMV, SPVG, SPVC, SPV2, SPCSV, SPMV, SPLCV and SPLCSPV [62,79,80]. SPBVA and SPBVB have collectively been assigned to the species SPPV [125] and their identity in South Africa was confirmed by conventional Sanger sequencing of amplified PCR products [81]. SPBVA, SPBVB, SPVC, SPVG and SPLCV were not as widespread as the rest of the viruses. The studies also revealed that DNA viruses occurred in mixed infections with RNA viruses. Yield reduction of multiple and co-infections under field conditions was evaluated and shown to vary between 28 and 100% depending on the sweet potato varieties and the viruses involved [62].

Tanzania was the second-largest producer of sweet potato in Africa 2016 [124]. Ninety-six symptomatic and asymptomatic sweet potato vines were collected from different locations in Tanzania between December 2012 to January 2013 [110]. Small RNAs were used for HTS. Nucleotide blast and blastx against the sequences in the GenBank database of the *de novo*-assembled contigs matched sequences of SPCSV, SPFMV, SPLCSPV, SPPV, and SPSMV-1. The presence of these viruses was confirmed using conventional molecular techniques. SPPV and SPSMV-1 were found widespread and co-infecting sweet potato plants in Tanzania [110].

### 3.4. Cassava

Cassava (*Manihot esculenta* subspecies *esculenta* Crantz; *Euphorbiaceae*), which is currently produced in 40 sub-Saharan African countries, was introduced to that region by Portuguese sailors from Brazil in the sixteenth century [126]. Although the leaves are used as food in some countries, what makes cassava very popular are its roots. According to the 2017 FAOSTAT, cassava was the number one root crop in sub-Saharan Africa ahead of yam and sweet potato with an annual root production exceeding 140 million tons [127]. Tubers, once processed are cooked using various traditional techniques, constitute the major source of carbohydrates in many households across sub-Saharan Africa. Besides being the most important staple food and a significant source of farm income, cassava has also enormous potential for industrial processing [128]. To turn that concept into reality will cause an increase in the demand for high-quality healthy cassava roots.

Africa’s average yield of cassava is far below the predicted yield under optimal conditions of 23.2 tons/ha [129]. The recorded average yield for the period 2015–2018 fluctuated between 8.9 and 9.2 tons/ha [130]. Viral diseases are an impending disaster to cassava production unless proper integrative control strategies are strictly observed. HTS has been used to unravel the etiology of some of the most damaging viral diseases of cassava in Cameroon [101], Comoros [66], Malawi [91], Mozambique [65], Tanzania [107,114], Togo [101] and Uganda [114].

The earliest records of HTS application on cassava are from 2010. Total RNA extracted from symptomatic leaves collected in Uganda, and Tanzania served as template in cDNA synthesis. A Subtractive hybridization designed to enrich the viral cDNA was performed prior to HTS on a pyrosequencing platform. Contig assembly was carried out using a commercial software CLC Genomics Workbench (CLCBio, Denmark). Blast analysis was performed using blastn. Alternately, metagenomic analysis was performed using MEGAN [131]. As the HTS data analysis showed gaps, specific primers were designed to enable the amplification of overlapping PCR products that enabled the construction of the missing portion of the viral genome sequences. The integrity of the 5’end of the genome was checked using rapid amplification of cDNA ends. CBSV was the only identified virus in the analyzed samples. Two distinct isolates, namely, CBSV Ugandan strain and CBSV Tanzanian strain were identified from this study. Sequence comparisons indicated 76% nucleotide identity across the genome and 57–77% protein identity [114]. The discovery of only ipomovirus sequences in that study put an end to the controversy over the causal agent of the cassava brown streak disease (CBSD). However, the sequence comparison results from that study provided enough evidence to consider each described isolate as a distinct species. CBSD was first reported in 1936 from Northeast Tanzania. The disease symptoms vary depending on the viral strain, cassava cultivar, environmental conditions and age of the plant at the time of infection. Symptoms that have been found associated with CBSD include plant root necrosis, radial root constrictions foliar chlorosis, brown streaks and lesions on the stem [132].

CBSD re-emerged at the turn of the 21st century. It has since spread through many East and Central African countries, causing considerable yield losses and jeopardizing the food security of subsistence farmers [132]. A cassava cutting with conspicuous symptoms of CBSD sampled in Nkhata Bay District in Malawi in 2013 was selected for HTS and data analysis. The ribo-depleted RNA of the sample under study was used as a template for double-stranded cDNA synthesis before library preparation for HTS on a MiSeq instrument. Data analysis was done using both CLC Workbench and Geneious (https://www.geneious). One well-supported contig that matched UCBSV genome sequence was selected and compared with all complete UCBSV genome sequences available in GenBank. This 9070-nucleotide contig constituted the genome sequence of an UCBSV isolate called MW-NB7_2013. This isolate was highly similar to an isolate from Tanzania (93.4% pairwise nucleotide identity) than to those previously reported from Malawi (86.9 to 87.0%) [91]. Similar studies have also been performed in Mozambique [65] and Comoros [66] and they strongly support the existence of new isolate lineages. The rampant spread of CBSD has triggered intense scientific mobilization to better understand the epidemiology, sequence diversity, host interactions and integrated control management of the disease.

## 4. Conclusions

The HTS for viromics studies performed on different host plants in the African continent has led to the detection of known and novel viruses. This observation is consistent in all hosts tested and across all the countries where the technology has been used. Molecular and serological detection methods remain relevant, especially in cases of novel viruses and viruses not previously reported. The unprecedented increase of novel viruses detected using HTS once again accentuates the need for a uniform and practical way of naming plant viruses. This call has been echoed several times in the scientific communities [133,134,135,136,137,138]. Plant-infecting viruses have been formerly named based on the host they were found to infect at the time of their discoveries and the type of symptoms they induced. Using such a method requires a thorough investigation of the virus symptoms in regards of the Koch’s postulates. However, the speed at which novel viruses are detected and reported outruns the follow-up studies on their biological properties. Furthermore, it has been proven through comprehensive HTS studies that plants are often infected with multiple viruses under natural field conditions. In some instances, co-infections with multiple unrelated viruses have turned into synergistic interactions that are characterized by an increase in viral replication or movement in the host plant, and the development of more severe symptoms. The revision of the current naming of plant-infecting viruses using a system independent of symptomatology may restore order and accuracy in that regard.

It is apparent that biological characterization of plant-infecting viruses has remained relevant throughout the history of plant virology. This is all the more true in this age of HTS and bioinformatics. In the past virus discovery was symptoms driven thus strongly relying on combinations of biological techniques, morphology and conventional targeted methods. The advent of HTS has resulted in an alteration of this pattern and preeminence has unintentionally been given to the molecular component. It is therefore critical to link molecular observations to their corresponding effects in the host plants. Taking multiple virus infections as an example, follow-up studies should be conducted to understand the host response to single and multiple infections. Plant health risk assessment should actually be carried out for every HTS detected virus more especially that there is evidence of the existence of cryptic [139,140,141] and even beneficial viruses [142]. The availability of scientific knowledge as such will enable plant health, policymakers and regulators to make correct decisions. Recommended literature on this topic includes Adams et al. [143] and Massart et al. [144].

The progress made in the field of plant virology throughout the African continent is quite tangible, albeit happening slowly. However, the nescience of the existence of plant-infecting viruses does not in any way prevent their spread. The detection of virus strains infecting maize and common bean in Africa that are similar to Asian strains is an obvious illustration of viruses readily spreading across continents probably through anthropogenic activities. The continual presence of plant-infecting viruses unknown to scientists on the African soil could, in the long term, have devastating consequences for agricultural production. Although the integration of HTS and bioinformatics tools in plant virology research projects in Africa will require substantial financial investments, its implementation and uptake in more countries than the case presently, will go a long way towards the development of effective and sustainable strategies to manage/control viral diseases infecting major crops. This will ensure food security for many food-insecure people on the continent.

## Figures and Tables

**Table 1:** Names, abbreviations and taxonomic status of viruses identified in Africa by HTS means.

Virus Name	Abbreviation	Genus	Family
African cassava mosaic virus	ACMV	*Begomovirus*	*Geminiviridae*
African eggplant yellowing virus	AeYV	*Polerovirus*	*Luteoviridae*
*Arctopus echinatus*-associated virus	AeaV	N/A	N/A
Barley virus G	BVG	*Polerovirus*	*Luteoviridae*
Barley yellow dwarf virus	BYDV	*Luteovirus*	*Luteoviridae*
Bean common mosaic virus	BCMV	*Potyvirus*	*Potyviridae*
Bean common mosaic virus strain blackeye lackeye cowpea mosaic	BCMV-BlCM	*Potyvirus*	*Potyviridae*
Bean common mosaic necrosis virus	BCMNV	*Potyvirus*	*Potyviridae*
Bean yellow disorder virus	BnYDV	*Crinivirus*	*Closteroviridae*
Cacao swollen shoot virus	CSSV	*Badnavirus*	*Caulimoviridae*
Cacao swollen shoot CD virus	CSSCDV	*Badnavirus*	*Caulimoviridae*
Cacao swollen shoot CE virus	CSSCEV	*Badnavirus*	*Caulimoviridae*
Cacao swollen shoot Ghana M virus	CSSGMV	*Badnavirus*	*Caulimoviridae*
Cacao swollen shoot Ghana N virus	CSSGNV	*Badnavirus*	*Caulimoviridae*
Cacao swollen shoot Ghana Q virus	CSSGQV	*Badnavirus*	*Caulimoviridae*
Cacao swollen shoot Ghana R virus	CSSGRV	*Badnavirus*	*Caulimoviridae*
Cacao swollen shoot Togo A virus	CSSTAV	*Badnavirus*	*Caulimoviridae*
Cassava brown streak virus	CBSV	*Ipomovirus*	*Potyviridae*
Chickpea chlorotic dwarf virus tomato strain	CpCDV-T	*Mastrevirus*	*Geminiviridae*
Coccinia mottle virus	CocMoV	*Ipomovirus*	*Potyviridae*
Cotton yellow mosaic virus	CYMV	*Begomovirus*	*Geminiviridae*
Cowpea aphid-borne mosaic virus	CABMV	*Potyvirus*	*Potyviridae*
Cowpea associated mycotymovirid 1	CPaMV-1	N/A	*Tymoviridae*
Cowpea mild mottle virus	CPMMV	*Carlavirus*	*Betaflexiviridae*
Cowpea mottle virus	CPMoV	*Gammacarmovirus*	*Tombusviridae*
Cowpea polerovirus 1	CPPV-1	*Polerovirus*	*Luteoviridae*
Cowpea polerovirus 2	CPPV-2	*Polerovirus*	*Luteoviridae*
Cowpea tombusvirid 1	CPTV-1	N/A	*Tombusviridae*
Cowpea tombusvirid 2	CPTV-2	N/A	*Tombusviridae*
Cucumber mosaic virus	CMV	*Cucumovirus*	*Bromoviridae*
Cucumber vein yellowing virus	CVYV	*Ipomovirus*	*Potyviridae*
Dioscorea bacilliform RT virus 3	DBRTV3	*Badnavirus*	*Caulimoviridae*
Dioscorea mosaic-associated virus	DMaV	*Sadwavirus*	*Secoviridae*
Grapevine endophyte alphaendornavirus	GEEV	*Alphaendornavirus*	*Endornaviridae*
Grapevine leafroll-associated virus 3	GLRaV-3	*Ampelovirus*	*Closteroviridae*
Grapevine rupestris stem pitting-associated virus	GRSPaV	*Foveavirus*	*Betaflexiviridae*
Grapevine virus A	GVA	*Vitivirus*	*Betaflexiviridae*
Grapevine virus E	GVE	*Vitivirus*	*Betaflexiviridae*
Grapevine virus F	GVF	*Vitivirus*	*Betaflexiviridae*
Groundnut rosette virus	GRV	*Umbravirus*	*Tombusviridae*
Groundnut rosette assistor virus	GRAV	N/A	*Luteoviridae*
Iris yellow spot virus	IYSV	*Orthotospovirus*	*Tospoviridae*
Ivy ringspot-associated virus	IRSaV	*Badnavirus*	*Caulimoviridae*
Johnsongrass mosaic virus	JGMV	*Potyvirus*	*Potyviridae*
*Lolium perenne*-associated virus	LpaV	N/A	N/A
Maize-associated betaflexivirus	MaBV	*Chordovirus*	*Betaflexaviridae*
Maize-associated pteridovirus	MaPV	*Pteridovirus*	*Mayoviridae*
Maize-associated totivirus	MATV	*Totivirus*	*Totiviridae*
Maize-associated totivirus-1-Tanz	MATV-1-Tanz	*Totivirus*	*Totiviridae*
Maize-associated totivirus-4-Tanz	MATV-4-Tanz	*Totivirus*	*Totiviridae*
Maize dwarf mosaic virus	MDMV	*Potyvirus*	*Potyviridae*
Maize chlorotic mottle virus	MCMV	*Machlomovirus*	*Tombusviridae*
Maize pteridovirus 1	MPtV-1	*Pteridovirus*	*Mayoviridae*
Maize streak virus	MSV	*Mastrevirus*	*Geminiviridae*
Maize yellow dwarf virus RMV2	MYDV-RMV2	*Polerovirus*	*Luteoviridae*
Maize yellow mosaic virus	MaYMV	*Polerovirus*	*Luteoviridae*
Moroccan watermelon mosaic virus	MWMV	*Potyvirus*	*Potyviridae*
Morogoro maize-associated virus	MMaV	*Alphanucleorhabdovirus*	*Rhabdoviridae*
Ornithogalum mosaic virus	OrMV	*Potyvirus*	*Potyviridae*
Ornithogalum virus 3	OV-3	*Potyvirus*	*Potyviridae*
Ornithogalum virus 5	OV-5	*Polerovirus*	*Luteoviridae*
*Panicum ecklonii*-associated virus	PeaV	N/A	N/A
Papaya mild mottle-associated virus	PaMMV	*Carlavirus*	*Betaflexiviridae*
Papaya mottle-associated virus	PaMV	*Carlavirus*	*Betaflexiviridae*
Papaya virus A	PaVA	*Allexivirus*	*Alphaflexiviridae*
Passiflora virus	PV	*Potyvirus*	*Potyviridae*
Peanut mottle virus	PeMoV	*Potyvirus*	*Potyviridae*
Pelargonium vein-banding virus	PVBV	*Badnavirus*	*Caulimoviridae*
Pepo aphid-borne yellows virus	PABYV	*Polerovirus*	*Luteoviridae*
Phaseolus vulgaris alphaendornavirus 1	PvEV-1	*alphaendornavirus*	*Endornaviridae*
Phaseolus vulgaris alphaendornavirus 2	PvEV-2	*alphaendornavirus*	*Endornaviridae*
Potato Virus Y	PVY	*Potyvirus*	*Potyviridae*
Plum bark necrosis stem-pitting-associated virus	PBNSPaV	*Ampelovirus*	*Closteroviridae*
Pumpkin polerovirus	PuPV	*Polerovirus*	*Luteoviridae*
Scallion mosaic virus	ScaMV	*Potyvirus*	*Potyviridae*
Sorghum mosaic virus	SrMV	*Potyvirus*	*Potyviridae*
Southern bean mosaic virus	SBMV	*Sobemovirus*	*Solemoviridae*
Southern cowpea mosaic virus	SCPMV	*Sobemovirus*	*Solemoviridae*
Soybean chlorotic blotch virus	SbCBV	*Begomovirus*	*Geminiviridae*
Squash chlorotic leaf spot virus	SCLSV	*Torradovirus*	*Secoviridae*
*Stipagrostis*-associated virus	SaV	N/A	N/A
Sudan watermelon mosaic virus	SuWMV	*Potyvirus*	*Potyviridae*
Sugarcane mosaic virus	SCM	*Potyvirus*	*Potyviridae*
Sugarcane streak Egypt Virus	SSEV	*Mastrevirus*	*Geminiviridae*
Sugarcane white streak Virus	SCWSV	*Mastrevirus*	*Geminiviridae*
Sweet potato chlorotic fleck virus	SPCFV	*Carlavirus*	*Betaflexiviridae*
Sweet potato chlorotic stunt virus	SPCSV	*Crinivirus*	*Closteroviridae*
Sweet potato feathery mottle virus	SPFMV	*Potyvirus*	*Potyviridae*
Sweet potato leaf curl virus	SPLCV	*Begomovirus*	*Geminiviridae*
Sweet potato leaf curl Sao Paulo virus	SPLCSPV	*Begomovirus*	*Geminiviridae*
Sweet potato leaf curl Uganda virus	SPLCUV	*Begomovirus*	*Geminiviridae*
Sweet potato mosaic virus	SPMV	*Begomovirus*	*Geminiviridae*
Sweet potato pakakuy virus	SPPV	*Badnavirus*	*Caulimoviridae*
Sweet potato virus 2	SPV2	*Potyvirus*	*Potyviridae*
Sweet potato virus C	SPVC	*Potyvirus*	*Potyviridae*
Sweet potato virus G	SPVG	*Potyvirus*	*Potyviridae*
Sweet potato symptomless virus 1	SPSMV-1	*Mastrevirus*	*Geminiviridae*
Telfairia mosaic virus	TelMV	*Begomovirus*	*Geminiviridae*
Tobacco mottle virus	TMoV	*Umbravirus*	*Tombusviridae*
Tomato chlorosis virus	ToCV	*Crinivirus*	*Closteroviridae*
Tomato leaf curl Uganda virus	ToLCUV	*Begomovirus*	*Geminiviridae*
Tomato mosaic virus	ToMV	*Tobamovirus*	*Virgaviridae*
Tomato spotted wilt orthotospovirus	TSWV	*Orthotospovirus*	*Tospoviridae*
Turnip yellows virus	TuYV	*Polerovirus*	*Luteoviridae*
Ugandan cassava brown streak virus	UCBSV	*Ipomovirus*	*Potyviridae*
Ugandan cassava brown streak virus Comoros	UCBSV-KM	*Ipomovirus*	*Potyviridae*
West African Asystasia virus 1	WAAV1	*Begomovirus*	*Geminiviridae*
Yam mosaic virus	YMV	*Potyvirus*	*Potyviridae*
Yam virus Y	YVY	N/A	*Betaflexiviridae.*
*Zea mays* chrysovirus 1	ZMCV1	*Alphachrysovirus*	*Chrysoviridae*
Zucchini shoestring virus	ZSSV	*Potyvirus*	*Potyviridae*

N/A: not assigned yet.

**Table 2:** Summary of published HTS studies conducted in African countries in relations to the detection and diagnosis of plant-infecting viruses.

Year ^!^	Country ^#^	Sequencing Platform	Country Where HTS Was Performed	Nucleic Acid Target	Host Plant	Plant-Infecting Virus Identified	References
2020	Zambia	Illumina MiSeq	South Africa	Total RNA	Common bean	SBMV*; BCMNV; BCMV; CABMV; CMV	[45]
2020	South Africa	Illumina NovaSeq	South Africa	Ribo-depleted RNA	Japanese plum (*Prunus salicina*)	PBNSPaV*	[46]
2020	South Africa	Illumina MiSeq	South Africa	Ribo-depleted RNA	*Ornithogalum thyrsoides*	OrMV; OV-3; OV-5**^+^**	[47]
2020	South Africa	Illumina NextSeq	South Africa	Ribo-depleted RNA	ivy (*Hedera helix*)	IRSaV**^+^**	[48]
2020	Zimbabwe	Illumina HiSeq	South Africa	Ribo-depleted RNA	*Dendranthema morifolium*	TSWV	[49]
2020	Zimbabwe	Illumina HiSeq	South Africa	Ribo-depleted RNA	Zucchini	ZSSV*	[50]
2020	Kenya	Illumina MiSeq	Kenya	Genomic DNA	Tomato	CpCDV-T**^+^**	[51]
2020	Kenya	Illumina MiSeq	Kenya	mRNA	Papaya	MWMV*; CPMMV*; PaMV**^+^** PMMaV**^+^**	[52]
2020	Kenya	Illumina HiSeq	South Africa	Ribo-depleted RNA	Papaya	MWMV; PaVA**^+^**	[53]
2020	Kenya; Rwanda; Tanzania; Uganda	Illumina MiSeq	Kenya	mRNA	Sweet potato	SPFMV	[54]
2020	Rwanda	Illumina MiSeq	USA	Ribo-depleted RNA	Maize	MCMV; SCMV; MSV; MYDV-RMV2; MYDV-like; MaYMV; BYDV; MATV**^+^**; MPtV-1**^+^**; ZMCV1*; MaBV**^+^**	[55]
2019	Tanzania	Illumina MiSeq	Kenya	Total RNA	Maize	MCMV; SCMV; MSV; MYDV-RMV; BYDV; MDMV; SrMV	[56]
2019	Tanzania	Illumina HiSeq	South Africa	Ribo-depleted RNA	Maize	MaPV**^+^**; MCMV; MSV; MaYMV	[57]
2019	Tanzania	Illumina HiSeq	South Africa	Ribo-depleted RNA	Maize	MATV-1-Tanz*; MATV-4-Tanz**+**;	[58]
2019	Tanzania	Illumina HiSeq	South Africa	Ribo-depleted RNA	Maize	MMaV**^+^**; MCMV; MaYMV	[59]
2019	Tanzania	Illumina HiSeq	South Africa	Ribo-depleted RNA	Maize	MaYMV*; MCMV	[60]
2019	South Africa	Illumina HiSeq	South Africa	Ribo-depleted RNA	Tomato	ToCV; Other viruses not mentioned	[61]
2019	South Africa	Illumina MiSeq	South Africa	Ribo-depleted RNA; RCA dsDNA	Sweet potato	SPFMV; SPVG; SPVC; SPV2; SPLCSPV; SPMV	[62]
2019	South Africa	454 pyrosequencing	USA	VANA	*Arctopus echinatus*; *Lolium perenne*; *Panicum ecklonii*; *Stipagrostis* sp.	AeaV; SaV; PeaV; LpaV	[63]
2019	Zimbabwe	Illumina HiSeq	South Africa	Ribo-depleted RNA	Garlic	IYSV	[64]
2019	Mozambique	Illumina MiSeq	South Africa	Ribo-depleted RNA	Cassava	CBSV; UCBSV	[65]
2019	Comoros	Illumina Hiseq	Switzerland	siRNAs; VANA; dsRNAs	Cassava	UCBSV-KM**^+^**; CBSV*	[66]
2019	Sudan	Illumina MiSeq	Switzerland	small RNAs	Cucurbit	CVYV	[67]
2019	Kenya	Illumina MiSeq	Kenya	Ribo-depleted RNA	Pumpkin	PuPV**^+^**; MWMV	[68]
2019	Kenya	Illumina MiSeq	Kenya	Total RNA	Groundnut	GRAV; Other viruses not mentioned	[69]
2019	Kenya	Illumina MiSeq	Kenya	mRNA	Common bean	BCMNV	[70]
2019	Kenya	Illumina HiSeq	South Korea	RCA dsDNA	Common bean	PVBV*; BCMNV	[71]
2019	Kenya	Illumina HiSeq	South Korea	Ribo-depleted RNA	Common bean; Cowpea	BCMNV; CABMV	[72]
2019	Kenya	Illumina MiSeq	Kenya	Ribo-depleted RNA	Passion fruit	CABMV	[73]
2019	Uganda	Illumina MiSeq	Kenya	Ribo-depleted RNA	Tannia (*Xanthosoma* sp.)	CMV*	[74]
2019	Uganda	Illumina MiSeq	Kenya	Total RNA	Passion fruit	PV**^+^**	[75]
2019	Nigeria	Illumina HiSeq	UK	Total RNA	*Dioscorea rotundata* (Yam)	YMV**^+^**; DBRTV3-[2RT]**^+^**; DBRTV3-[3RT]**^+^**	[76]
2019	Nigeria; Ghana	Illumina HiSeq	UK	Total RNA	*Dioscorea rotundata*	YVY**^+^**; YMV; DMaV; Yam badnavirus	[77]
2018	Zimbabwe	Illumina MiSeq	South Africa	Ribo-depleted RNA	Tomato	ToMV	[78]
2018	South Africa	Illumina MiSeq	South Africa	RCA dsDNA	Sweet Potato	SPLCV**^+^**; SPLCSPV; SPMV	[79]
2018	South Africa	Illumina MiSeq	South Africa	Ribo-depleted RNA; RCA dsDNA	Sweet potato	SPCSV; SPFMV; SPVC; SPVG; SPMV; SPLCSPV	[80]
2018	South Africa	Illumina MiSeq	South Africa	siRNAs	Sweet potato	SPPV*	[81]
2018	Kenya	Illumina Hiseq	South Korea	Ribo-depleted RNA	Sweet potato	SPCFV; SPFMV; SPVC; SPCSV	[82]
2018	Kenya	Illumina Hiseq	South Korea	Ribo-depleted RNA	Groundnut	GRV	[83]
2018	Kenya	Illumina MiSeq	Kenya	Total RNA	Common bean	PvEV-1**^+^**; PvEV-2**^+^** BCMNV, CMV	[84]
2018	Uganda	Illumina MiSeq	Kenya	mRNA	Cowpea	CABMV	[85]
2018	Tanzania	Illumina HiSeq	Switzerland	siRNAs	Common Bean	Umbraviruses**^+^**; ToLCUV*; PeMoV; BnYDV; TMmV SBMV*; BCMNV; BCMV; PvEV-1; PvEV-2; CpMMV; CMV; CABMV	[86]
2018	Uganda; Kenya; Rwanda; Tanzania	Illumina Hiseq	USA	mRNA	Maize	MYDV-like polerovirus*; East African JGMV; SCMV; MCMV	[87]
2018	Kenya	Illumina MiSeq	Kenya	Total RNA	Maize; Sorghum; Napier grass	Hubei Poty-like virus 1; BVG; ScaMV; MCMV; SCMV; MSV; MYDV-RMV; JGMV	[88]
2018	Côte d’Ivoire; Ghana	Illumina HiSeq Illumina MiSeq	France	RCA dsDNA	Cacao	CSSCEV**^+^**; CSSGMV**^+^**; CSSGNV**^+^**; CSSGQV**^+^**; CSSGRV**^+^**; CSSV; CSSTAV; CSSCDV	[89]
2017	Benin; Mali	Illumina MiSeq	Germany	Ribo-depleted RNA	*Solanum aethiopicum*; Pepper (*Capsicum spp*.)	AeYV**^+^**	[90]
2017	Malawi	Illumina MiSeq	Germany	Ribo-depleted RNA	Cassava	UCBSV	[91]
2017	Tanzania	Illumina HiSeq	Switzerland	siRNAs	Common bean	PvEV-1; PvEV-2; CPMMV	[92]
2017	Sudan	Illumina HiSeq	Switzerland.	siRNAs	Cucumber	SuWMV**^+^**	[93]
2017	South Africa	Illumina HiSeq	South Africa	Ribo-depleted RNA	Zucchini	PABYV**^+^**	[94]
2017	South Africa	Illumina HiSeq	South Africa	Ribo-depleted RNA	Potato	PVY	[95]
2016	South Africa	Illumina HiSeq	South Africa	Total RNA	Cabbage	TuYV*	[96]
2016	South Africa	Illumina HiSeq	South Africa	Ribo-depleted RNA	Pattypan; Zucchini	ZSSV**^+^**; MWMV	[97,98]
2016	Sudan	Illumina HiSeq	Switzerland	siRNAs	Squash	SCLSV**^+^**	[99]
2016	Sudan	Illumina HiSeq	Switzerland	siRNAs	Wild cucurbit	CocMoV	[100]
2016	Cameroon; Togo	Illumina HiSeq	USA	RCA dsDNA	Cassava	SbCBV*; WAAV1*; ACMV	[101]
2016	Cameroon	Illumina MiSeq	USA	RCA dsDNA	Pumpkin	TelMV**^+^**	[102]
2016	Benin	Illumina MiSeq	USA	RCA dsDNA	Cotton	CYMV**^+^**	[103]
2016	Burkina Faso	454 pyrosequencing	USA	VANA	Cowpea	CPPV-1**^+^**; CPPV-2**^+^**; CPTV-1**^+^**; CPTV-2**^+^**; CPaMV-1**^+^**; SCPMV*; BCMV-BlCM; CPMoV; CABMV	[104]
2015	South Africa	Illumina HiSeq	South Africa	dsRNAs	Grapevine	GLRaV-3 isolate GH24	[105]
2015	South Africa	Illumina HiSeq	South Africa	dsRNAs	Grapevine	GVF; GLRaV-3	[106]
2015	Tanzania	Illumina MiSeq	Kenya	Ribo-depleted RNA	Cassava	CBSV; UCBSV**^+^**	[107]
2015	Ethiopia	Illumina MiSeq Illumina HiSeq	UK	Total RNA	Maize	MCMV*; SCMV	[108]
2014	Rwanda	Illumina MiSeq	UK	Total RNA	Maize	MCMV*; SCMV*	[109]
2014	Tanzania	Illumina HiSeq	Switzerland	siRNAs	Sweet potato	SPPV; SPSMV-1; SPLCUV; SPCSV; SPFMV; SPLCSPV**^+^**; SPCFV	[110]
2014	Egypt	Illumina HiSeq; Roche 454 GS FLX Titanium	USA	siRNAs; VANA	Sugarcane	SSEV; SCWSV**^+^**	[111]
2013	Kenya	Roche 454 GS-FLX+	UK	Total RNA; dsRNAs	Maize	MCMV*; SCMV	[112]
2012	South Africa	Illumina HiSeq	South Africa	dsRNAs	Grapevine.	GEEV**^+^**	[113]
2010	Uganda; Tanzania	Roche GS-FLX	UK	Total RNA	Cassava	CBSV; UCBSV	[114]
2010	South Africa.	Illumina	South Africa	dsRNAs	Grapevine	GVE**^+^**; GRSPaV; GVA; GLRaV-3	[115]

**^!^**: Year of publication of the study; **^#^:** Country where the study was conducted or where the studied material originated from; *: First report of the virus in the respective country; **^+^**: Novel or previously unknown strain or species; RCA dsDNA: Rolling circle amplification double stranded DNA; siRNAs: virus-derived small interfering RNAs; VANA: Virion-associated nucleic acid; dsRNAs: Double-stranded RNAs.
